# Towards a practical implementation of X-ray ghost imaging with synchrotron light

**DOI:** 10.1107/S205225251800711X

**Published:** 2018-06-07

**Authors:** Daniele Pelliccia, Margie P. Olbinado, Alexander Rack, Andrew M. Kingston, Glenn R. Myers, David M. Paganin

**Affiliations:** a Instruments and Data Tools Pty Ltd, Victoria 3178, Australia; b School of Science, RMIT University, Victoria 3001, Australia; c European Synchrotron Radiation Facility, 38043 Grenoble, France; d Department of Applied Mathematics, Research School of Physics and Engineering, The Australian National University, Canberra ACT 2601, Australia; e CTLab: National Laboratory for Micro Computed-Tomography, Advanced Imaging Precinct, The Australian National University, Canberra ACT 2601, Australia; f School of Physics and Astronomy, Monash University, Victoria 3800, Australia

**Keywords:** X-ray imaging, X-ray ghost imaging, X-ray speckle, coherence, computational X-ray imaging, hard X-rays, point-spread function

## Abstract

An experimental procedure for transmission X-ray ghost imaging using synchrotron light is presented. Hard X-rays from an undulator were divided by a beamsplitter to produce two copies of a speckled incident beam. Both beams were simultaneously measured on an indirect pixellated detector and the intensity correlation between the two copies was used to retrieve the ghost image of samples placed in one of the two beams, without measuring the samples directly. Aiming at future practical uses of X-ray ghost imaging, the authors discuss details regarding data acquisition, image reconstruction strategies and measure the point-spread function of the ghost-imaging system. This approach may become relevant for applications of ghost imaging with X-ray sources such as undulators in storage rings, free-electron lasers and lower-coherence laboratory facilities.

## Abstract 2

A practical experimental procedure for transmission X-ray ghost imaging (XGI) using synchrotron light is presented. The authors demonstrate the method, discuss data acquisition and analysis, and measure the point-spread function of an XGI system. The generalization of the methods for future experiments is also discussed.

## 1. Introduction   

Imaging methods may be broadly categorized as either direct or indirect. Here, direct refers to any form of X-ray imaging in which the acquired image or images exhibit forms, shapes or structures directly related to the morphology of the object. Examples of direct imaging in an X-ray context include absorption-contrast X-ray imaging, X-ray interferometry, analyser-based X-ray phase-contrast imaging, Zernike phase-contrast X-ray imaging and propagation-based X-ray phase contrast. Methods for indirect X-ray imaging, in which the registered data bear no direct resemblance to the sample, include X-ray crystallography, coherent diffractive X-ray imaging, inline X-ray holography and X-ray ghost imaging.

The field of ghost imaging, which originated in visible-light quantum optics (Klyshko, 1988 ▸; Belinskii & Klyshko, 1994 ▸; Pittman *et al.*, 1995 ▸; Strekalov *et al.*, 1995 ▸; Bromberg *et al.*, 2009 ▸; Katz *et al.*, 2009 ▸; Erkmen & Shapiro, 2010 ▸; Shapiro & Boyd, 2012 ▸; Shirai, 2017 ▸), has a rich history which will not be reviewed here. Rather, our focus in the present paper is on the translation of the ghost-imaging concept into the classical X-ray domain. Indeed, with only four published papers at the time of writing (Yu *et al.*, 2016 ▸; Pelliccia *et al.*, 2016 ▸; Schori & Shwartz, 2017 ▸; Zhang *et al.*, 2018 ▸), the field of X-ray ghost imaging is still in its absolute infancy and has much scope for optimization and further development.

With a view to developing an intuitive understanding of the essence of the ghost-imaging concept, let us begin with an idea that is ubiquitous in physics, mathematics and engineering, namely the concept of Fourier series. The principle of Fourier series is that a function can be expanded as a linear combination of sinusoidally oscillating functions with different frequencies, which act as basis functions. What is often much less appreciated is that noise can form a basis too; therefore, it is possible to express functions as a sum of different noise maps (see *e.g.* Ceddia & Paganin, 2018*a* ▸,*b* ▸), in a conceptually similar manner to building functions as a sum of sinusoids in a Fourier series. Applied to imaging, this becomes the essence of the ghost-imaging concept. Here, ‘noise maps’ may equate for instance to speckle fields, or to shot-noise maps, while the functions that are to be decomposed become images that are to be synthesized by superposing noise maps.

Stated more precisely, the essence of the classical ghost-imaging concept (together with the closely related notion of computational imaging), may be captured in two ideas. (i) Spatially random fields, such as speckle fields, may be used as a basis in the sense that the intensity transmission function of a thin object can be approximately expressed as a linear combination of such speckle fields; (ii) the coefficients, in the expansion of an object’s transmission function in terms of the speckle basis, may be obtained using an ensemble of single-pixel (‘bucket’) correlation measurements of the total intensity transmitted by the sample, for each known (or measured) illuminating speckle field. When the illuminating speckle fields are measured at the same time as the bucket signals, we speak of ghost imaging, while if the illuminating speckle fields are known then we speak of computational ghost imaging.

The speckled-intensity basis elements may be spontaneously generated using, for example, the X-ray photon shot noise from individual electron bunches (Pelliccia *et al.*, 2016 ▸), or may be deterministically created using, for example, transmission of a plane wave through a scattering object (Yu *et al.*, 2016 ▸). In either case, it is interesting that ghost imaging is able to ‘synthesize signals as a superposition of noise’.

The current literature on experimental realizations of X-ray ghost imaging is sparse. With the exception of the very recent work by Zhang *et al.* (2018 ▸), all published X-ray ghost-imaging reconstructions (Yu *et al.*, 2016 ▸; Pelliccia *et al.*, 2016 ▸; Schori & Shwartz, 2017 ▸) are one-dimensional. The paucity of the existing literature on experimental realizations of X-ray ghost imaging is an obvious indicator of avenues for future work. Indeed, it is our view that significant further work is needed to clarify whether X-ray ghost imaging is merely an interesting novelty, or has the capacity to develop into a technique of genuine utility and enduring value.

The early experimental realizations of X-ray ghost imaging have followed two approaches: (i) using a beamsplitter to create two copies of the speckled incident beam, with the sample located in one of the beams (Pelliccia *et al.*, 2016 ▸; Schori & Shwartz, 2017 ▸) or (ii) measuring the speckled beam directly without splitting, and repeating the cycle of measurements twice, with and without the sample (Yu *et al.*, 2016 ▸; Zhang *et al.*, 2018 ▸). The second approach is well suited when the speckled beam can be accurately reproduced, and has the advantage of avoiding X-ray optics to split the beam. The first approach on the other hand, dispenses with the need to control the incident speckles, as both beams are measured simultaneously. This is an evident advantage when the speckles cannot be controlled with high accuracy, or when they are generated through a genuinely random process, such as electron shot noise in an undulator when using a synchrotron source (Pelliccia *et al.*, 2016 ▸). Note also that the signal-to-noise ratio (SNR) of shot-noise-induced speckles is limited by the bunch brightness, whereas the SNR of scatterer-induced speckles may be controlled by varying the nature of the scatterer, or the energy of the X-rays, and can be increased significantly by prolonging the exposure time.

A key feature of ghost imaging is its robustness with respect to non-correlated fluctuations in the two arms of a ghost-imaging setup (Meyers *et al.*, 2007 ▸). Since it relies on intensity correlations, the ghost signal is unaffected by such non-correlated post-beamsplitter random perturbations in the split signals. Another attractive feature, which is especially appealing when using ionizing radiation, is the possibility of dose reduction on the sample (Yu *et al.*, 2016 ▸; Pelliccia *et al.*, 2016 ▸; Li *et al.*, 2018 ▸; Zhang *et al.*, 2018 ▸).

Ghost imaging is also potentially relevant for future applications with free-electron lasers (FELs) and other brilliant sources of pulsed X-rays. The high spatial coherence of these sources permits a high degree of control on the speckled illumination on the sample. Along with the development of suitable, high-frame-rate detectors (Hatsui & Graafsma, 2015 ▸), ghost imaging may represent an alternative way to overcome radiation damage for structural biology experiments (Spence, 2017 ▸). At the same time, a high degree of spatial coherence can also boost the efficacy of intensity correlation techniques to gather information about the sample. However, we emphasize that coherence is not a requirement of the method, making translation to lower-coherence laboratory sources, such as was achieved in the recent investigation of Schori & Shwartz (2017 ▸), an interesting avenue for further work.

A key motivation for studying X-ray ghost imaging, within an X-ray context, is the previously mentioned possibility it may give for a reduced dose. While the data available so far seem to suggest that dose reduction may be possible in a conventional imaging setting (Zhang *et al.*, 2018 ▸), it is currently not clear whether the same idea could benefit future application to free-electron lasers, to avoid sample destruction.

Here, we present a two-dimensional, high-resolution, experimental realization of X-ray ghost imaging, with a higher number of pixels in each transverse dimension than the previously cited experimental studies. Two different methods of ghost-image reconstruction are considered, together with a study comparing spatial resolution in the two methods. Our approach is to split the X-ray beam to produce two correlated copies of the speckled illumination. We believe this method offers a practical avenue for X-ray ghost imaging. (i) It is scalable to tomography (Kingston *et al.*, 2018 ▸) and even finer spatial resolutions. (ii) It is amenable to highly parallel geometries, in which ghost images of many objects may be acquired simultaneously.

We close this introduction with a brief outline of the remainder of the paper. Section 2 gives an outline of the X-ray synchrotron-based experimental setup and measurement process for two-dimensional X-ray ghost imaging. Section 3 presents our reconstructed two-dimensional images, of both a perforated lead-sheet stencil and the tungsten filament of an incandescent light globe. Section 4 discusses resolution, robustness, parallelization and computational X-ray ghost imaging. This final section also includes some considerations regarding possible future developments in the currently infant field of X-ray ghost imaging.

## 2. Experiment   

### 2.1. Experimental setup   

**Figure 1:**
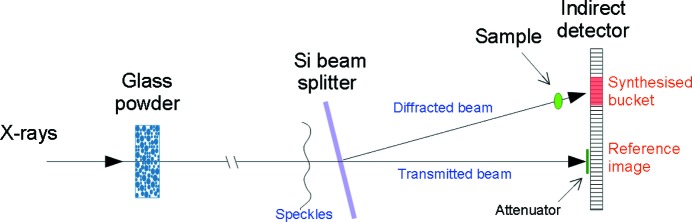
Schematic diagram of the experimental setup.

The experiments were carried out at beamline ID19 of the ESRF in Grenoble (France). Pink radiation from a so-called single-harmonic undulator, with a mean energy of 26.3 keV, was focused by a stack of compound refractive lenses to a focal spot of about 5.5 mm diameter at the sample. To produce variable (and controllable) speckles in the beam we inserted a 1 cm thick perspex container filled with glass powder in the beam, 5.8 m upstream of the sample. The powder (Oberflächentechnik Seelmann, Germany) was composed of grains, irregular in shape, with a typical size distributed in the range 200–1000 µm. Propagation-based phase-contrast (Snigirev *et al.*, 1995 ▸) from these beads generated a speckle pattern on the sample, that could be controlled by raster scanning the perspex container in the transverse plane. The contrast of the speckle pattern generated in this way was about 14%, as measured in a region around the centre of the beam. A silicon crystal beamsplitter, placed 20 cm upstream of the sample, was used to produce two copies of the beam *via* Laue diffraction (transmission geometry) from the (220) planes of the silicon. The primary beam was mostly transmitted by the Si wafer (see Fig. 1 ▸), thus creating two identical copies of the beam, though with highly unbalanced intensities. Both beams were then recorded by the same pixel array detector (scintillator lens-coupled to a FReLoN camera), placed immediately downstream of the sample. The effective pixel size of the camera was 30 µm.

**Figure 2:**
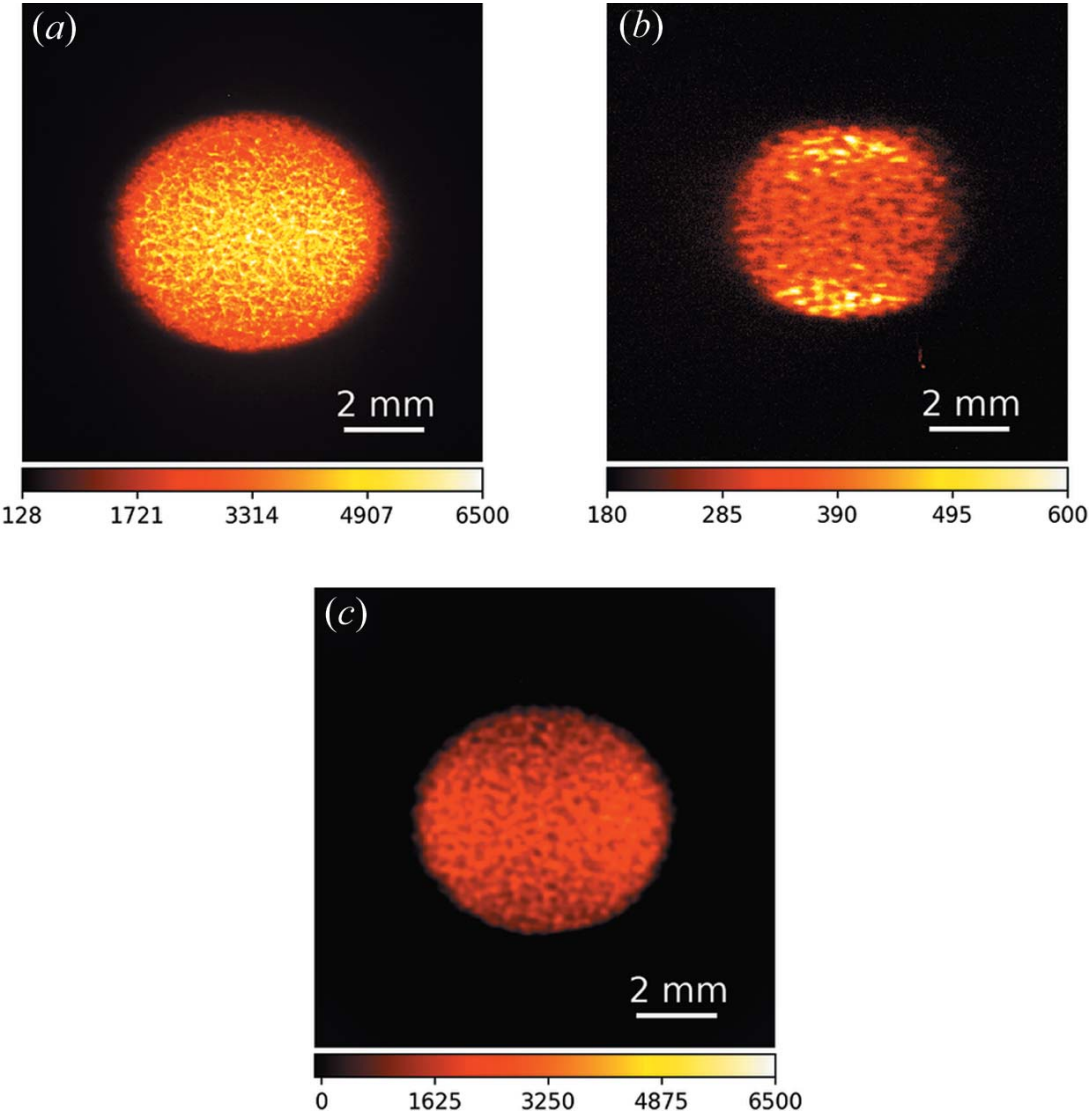
(*a*) Image of the primary beam on the FReLoN camera acquired with 2 s exposure time. The beam was attenuated by stacking a 500 µm thick Cu foil and a 500 µm thick GaAs wafer to avoid saturation. (*b*) Corresponding image of the diffracted beam. No attenuator was placed in the diffracted beam path. (*c*) Blurred version of the image in (*a*) to highlight the similarities in the speckle pattern distribution between the direct and the diffracted beam.

The attenuated image of the primary beam, with the glass-beads slab in place, is shown in Fig. 2 ▸(*a*). An attenuator composed of a 500 µm thick Cu foil and a 500 µm thick GaAs wafer was inserted in the primary beam to avoid saturation and protect the camera during the prolonged exposures. The corresponding image of the diffracted beam (cropped from the same frame of the camera) is shown in Fig. 2 ▸(*b*). The image was acquired with 2 s exposure time. Notice the different intensity of the two beams, with the diffracted beam being much weaker than the primary beam. The ratio of the average intensities in a region around the centre of the diffracted and direct beam was estimated by successive measurements with and without attenuators to be 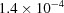.

Three main features of our ghost-imaging configuration based on an X-ray beam splitter need some more discussion. (i) Owing to unavoidable vibration of the silicon crystal, the image of the diffracted beam appears blurred when compared with the primary beam. To facilitate comparison between the two copies, and understand the scale of the vibration, we blurred the primary beam image and show the result in Fig. 2 ▸(*c*). The blurring was performed using a Gaussian kernel with standard deviation 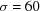 µm (2 pixels), which can be taken as the approximate spatial extent of the vibration of the Si wafer. This vibration-induced blurring is likely to locally decrease the speckle-to-speckle correlation between reference and bucket beam. This in turn is expected to decrease the efficiency of the ghost-imaging formation process, while leaving the resolution of the system unaffected. The issue is discussed in more detail in §4. Note that the blurring is presented here only for the sake of explaining the difference between a direct and diffracted beam. The ghost-imaging reconstruction described in the next section was performed without introducing this artificial blurring in the direct beam. (ii) The diffracted beam appears to be slightly compressed, probably because of some strain generated in the crystal mount, with more intensity being diffracted around the top and bottom edge of the beam. As we will see in §3.2, this generates some distortion in the ghost images, when compared with the original images, in the proximity of these edges. (iii) The diffracted beam has a much narrower energy bandwidth than the primary beam. Again, ghost imaging can be realized to the extent to which a speckle correlation is maintained between the two beams, regardless of the energy difference. This aspect has been demonstrated in a more general way by Aspden *et al.* (2015 ▸) where ghost imaging was demonstrated using photons of different wavelengths (visible and infrared).

### 2.2. Acquisition protocol   

The sample was inserted in the diffracted beam rather than the direct beam. We used this configuration to ensure the sample was illuminated by the weaker beam. Given our estimated intensity ratio, and assuming the measured counts to be proportional to the number of photons (this is nearly a monochromatic case), we expect that the sample receives 0.014% of the photons in the reference beam. The variable speckle illumination was obtained by raster scanning the glass-beads slab in the transverse plane. The range of the raster scan was 150 × 90 mm (*H* × *V*), with a step size of 750 × 500 µm (*H* × *V*). The step size was chosen to be about three times larger than the typical speckle size, to ensure that images taken at neighbouring positions along the scan were nearly independent. The typical speckle size is in fact much smaller than the average (or median) size of the glass beads, being determined by the wave propagation parameters of the phase-contrast image generated at the sample. The average speckle size, as discussed in detail in §4.1, is the point-spread function of the ghost-imaging system. In our case, the associated resolution is about 125 µm (see §4).

A speckle image was acquired at each position of the glass-beads slab, with 2 s exposure time. The bucket signal was then synthesized by summing the number of counts in an area of 200 × 180 pixels comprising the diffracted beam. The reference image at each position was generated by cropping an area of 260 × 230 pixels centred around the primary beam from the raw (attenuated) camera image. We acquired a total of 5000 frames for each ghost-imaging reconstruction. Note that, unlike our previous realization of direct X-ray ghost imaging (Pelliccia *et al.*, 2016 ▸), in this case the time structure of the synchrotron light is irrelevant. In our previous work we used the natural speckles arising from the shot noise in the X-ray emission of individual electron bunches. Here the glass beads are responsible for the speckled illumination, in a procedure that can be used in pulsed and continuous sources alike.

Owing to the periodic electron injections into the ESRF storage ring, a low-frequency time structure (with a period of 1 h) was present in the intensity signal. To prevent this feature affecting the ghost-imaging reconstruction, the data (both bucket signal and reference images) were Fourier filtered to remove such low frequency components. The filter was a simple low-pass designed to remove all frequencies below 5% of the Nyquist (temporal) frequency, and therefore discard all slow variations of the beam intensity owing to the injections.

## 3. Results   

### 3.1. Reconstruction   

Conventional ghost-imaging reconstruction can be obtained with the formula (Bromberg *et al.*, 2009 ▸; Katz *et al.*, 2009 ▸): 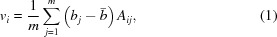
where 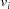 is the *i*th pixel of the (rasterized) ghost image **v**, written as the superposition of the corresponding pixels of the measured speckle images 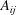 (*i*th pixel of the *j*th measurement). Each term of the superposition is weighted by the corresponding bucket signal 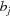 subtracted by its mean 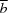. The total number of images used in the process is *m*. In our experiment 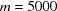. The ghost image **v** consists of 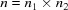 pixels. Denoting by **A** the 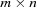 matrix of reference images, the bucket signal is 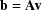.

Equation (1) can be written in the compact form: 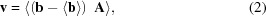
where 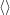 denotes ensemble average.

Before we continue, we return to the comments made in the introduction, that a key concept in ghost imaging is the idea of noise-maps (such as speckle fields) being a basis out of which optical images may be synthesized. This is precisely what equation (1) achieves, since it synthesizes the ghost images in terms of a linear combination of speckle images. This idea of speckle fields as a basis has been recently investigated by Ceddia & Paganin (2018*a* ▸,*b* ▸) and Gureyev *et al.* (2018 ▸).

The use of random speckle images (defined mathematically by the random measurement matrix 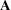) is however not optimal: to attain a good signal-to-noise ratio (SNR) a large number of measurements is generally required, *i.e.* 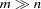, which makes the basic protocol unsuitable for applications demanding low dose. A better basis is made of orthonormal rather than merely linearly independent vectors, therefore the quality of the ghost image recovery depends on how well the rows of the random measurement matrix approximate an orthonormal basis (see Appendix *A* [App appa] for more detail). It is also worth noting that genuinely random illumination can be hard to produce in practice. In our case, we took care to scan the glass-beads slab by a transverse step size that was much larger than the transverse speckle size, however, residual correlations were still present.

To overcome such limitations, several approaches are currently used. Compressive ghost-imaging speeds image recovery using ideas and techniques of compressive sensing (Katz *et al.*, 2009 ▸), improving image recovery by identifying an orthonormal basis in which the image to be recovered is sparse (Candés *et al.*, 2006 ▸; Candés & Wakin, 2008 ▸; Katz *et al.*, 2009 ▸). In an alternative approach, commonly used in single-pixel cameras, image recovery can be much improved if one starts from an orthonormal measurement matrix (or sensing matrix) in the first place. A common choice is the Hadamard matrix **H** implementing Hadamard–Walsh functions *via* a spatial light modulator (see for instance, Clemente *et al.*, 2013 ▸, and Appendix *B* [App appb]).

As will be clear by looking at the results in §3.2, the limitations we discussed above severely affect the ghost-imaging reconstruction using equations (1) or (2). To improve the quality of the reconstructed images, we developed two independent strategies. In a first approach, inspired by orthonormal matrices such as **H**, we performed an effective orthogonalization of the background-subtracted speckle fields using the QR decomposition of the matrix **A**: 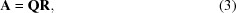
where, 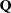 is the orthogonal matrix we seek, and 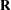 is an upper triangular matrix.

Once the QR decomposition has been found, the relevant transformation for the bucket signal can be computed (the mathematics involved in this process is explained in Appendix *B* [App appb]) to obtain a modified bucket 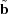 whose average is zero. The ghost-imaging reconstruction can then be obtained using equation (2) with 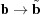 and 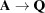 (note that 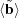 = 0): 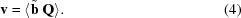
Reconstructing a ghost image using equation (4) has two main advantages over the conventional formula in equation (2). First, it guarantees better use of the information, as the new measurement matrix is now composed of orthogonal rows. Second, since the QR decomposition scrambles the basis, the typical speckle size of the orthogonal measurement matrix **Q** becomes effectively smaller than the typical size of the physical speckles used in the measurement. Hence, the resolution of the reconstructed ghost image is no longer limited by the real speckle size (*cf.* Oh *et al.*, 2013 ▸; Sprigg *et al.*, 2016 ▸), at the price of increased noise in the reconstruction.

Notably, one could also overcome the noise problem, by noting that for under-constrained problems (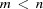) one could perform a QR decomposition multiple times by permuting the rows of **A** (and, correspondingly, of **b**) each time. In practice one obtains a different reconstruction for each permutation, but the difference in the reconstructions is mostly in the noise background. By averaging multiple reconstructions (using always the same data, hence not increasing radiation dose), one could reduce noise and increase resolution of the reconstruction at the same time.

A different approach, which may be useful in the presence of noisy data, is to perform an iterative refinement of the basic reconstruction obtained using equation (2) with a gradient descent method (Kingston *et al.*, 2018 ▸). The iterative refinement is iterated until a suitable convergence criterion is achieved (Huang *et al.*, 2018 ▸). In the next section we will show results obtained using all of the methods described.

### 3.2. Experimental results   

**Figure 3:**
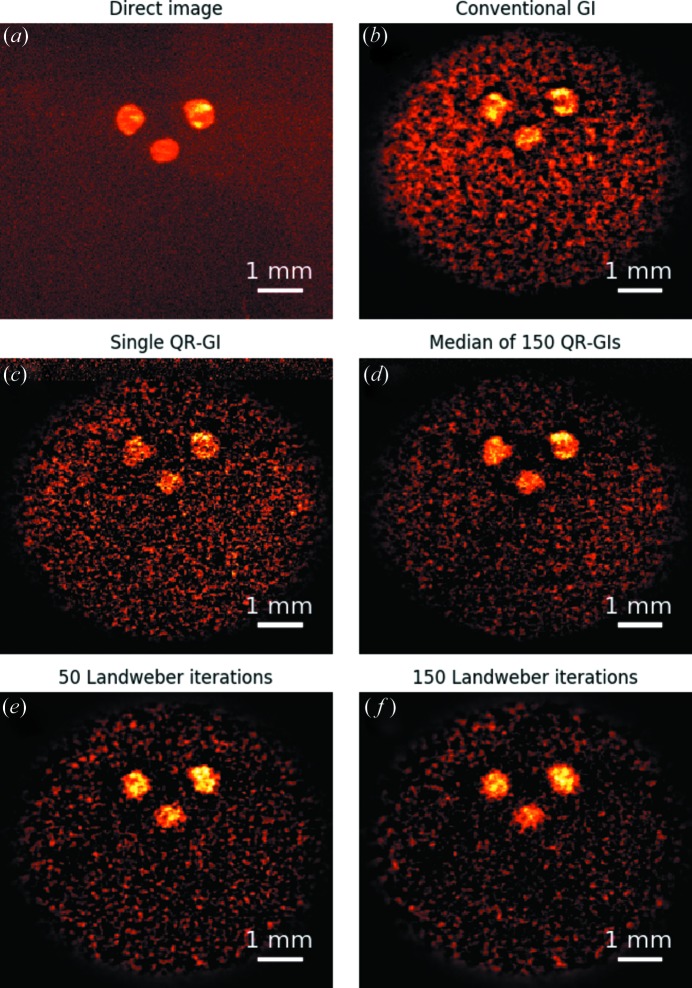
Measurement of the stencil sample. (*a*) Direct image of the sample when illuminated by one realization of the speckle pattern. (*b*) Conventional ghost-imaging reconstruction using 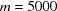 measurements. (*c*) Ghost-imaging reconstruction after QR decomposition of the measurement matrix, obtained using the same measurement as the previous case. (*d*) Median image of 150 ghost images obtained by QR decomposition of the randomly permuted measurement matrix. (*e*) Image obtained by iterative refinement of the image in (*b*) using 50 Landweber iterations. (*f*) Corresponding refined image using 150 Landweber iterations.

The first sample we imaged was a stencil obtained by drilling three holes in a lead sheet. The direct image of the sample in the diffracted beam (before synthesizing the bucket signal) is shown in Fig. 3(*a*). The ghost image, obtained using equation (2) with *m* = 5000, is shown in Fig. 3 ▸(*b*). Note that the ghost image size is equal to the reference image size of 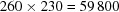 pixels. Therefore, we acquired a little more than 8% of the Nyquist sampling. The standard ghost-imaging reconstruction clearly reproduces the sample features albeit at a reduced resolution, as dictated by the speckle size, and is unaffected by the previously mentioned vibration in the bucket beam. Significant background noise is also present as a consequence of the limited number of measurements and the camera noise.

As discussed in the previous section, resolution can be improved *via* QR decomposition of the measurement matrix. The result of this operation [see equation (4) and the discussion in Appendix *B* [App appb]] is shown in Fig. 3(*c*) ▸. Resolution is improved, to the detriment of noise which is increased. This trade-off is consistent with the noise-resolution uncertainty principle (Gureyev *et al.*, 2016 ▸). By repeating the QR decomposition 150 times (each time performing a random permutation of the rows of the measurement matrix 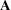 and, correspondingly, of the bucket values 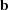) and taking the median of those images, the map in Fig. 3 ▸(*d*) can be synthesized. This last image is comparable to the conventional reconstruction in terms of noise and displays higher resolution.

The last two panels of Fig. 3 ▸ shows the result of the Landweber iterative refinement of the basic reconstruction in Fig. 3 ▸(*b*), using 50 and 150 iterations, respectively. The iterative refinement is helpful in reducing the background noise, while producing a somewhat smoother reconstruction.

**Figure 4:**
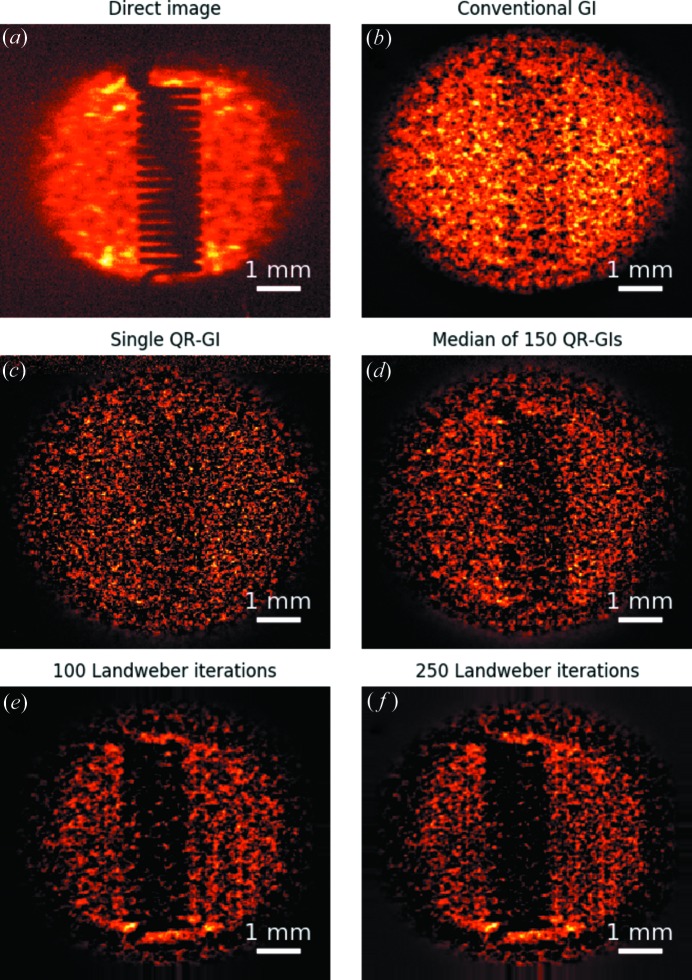
Measurement of the tungsten coil. (*a*) Direct image of the sample when illuminated by one realization of the speckle pattern. (*b*) Conventional ghost-imaging reconstruction using 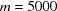 measurements. (*c*) Ghost-imaging reconstruction after QR decomposition of the measurement matrix, obtained using the same measurement as the previous case. (*d*) Median image of 150 ghost images obtained by QR decomposition of the randomly permuted measurement matrix. (*e*) Image obtained by iterative refinement of the image in (*b*) using 100 Landweber iterations. (*f*) Corresponding refined image using 250 Landweber iterations.

The corresponding set of images for the second sample, a tungsten coil, is shown in Fig. 4 ▸. The map obtained by the median of 150 ghost images obtained after QR decomposition shows a marked improvement over the conventional ghost image, which reflects the advantage of the QR decomposition method by optimizing the use of the available information. As before, the Landweber refinement further improves the contrast of the reconstructed image.

When compared with the stencil reconstruction, the ghost image of the coil looks noisier. Both images have been reconstructed using the same number of measurements. The difference is to be found in the sample extent compared to the beam size. The stencil sample is effectively composed of three holes only, whose size is relatively small compared with the beam. Hence, variations in the speckles’ position amount to relatively large excursions of the bucket signal. Conversely, the bucket signal after the coil sample will vary comparably much less, as it receives contributions from most of the beam size. Therefore we expect that the ghost-imaging procedure is much more sensitive when reconstructing the stencil, as opposed to the tungsten coil.

Finally, as anticipated in §2.1, owing to the beam compression by the silicon beamsplitter, the ghost images appear slightly distorted in the proximity of the top and bottom edge of the beam. This is somewhat more evident observing the coil images in Fig. 4 ▸, while, however, being present to the same extent in the ghost image reconstructions of both samples.

## 4. Discussion   

### 4.1. Point-spread function of the ghost-imaging system   

The standard ghost-imaging formula in equation (1) considers the ensemble of linearly independent random speckle images as a basis from which to synthesize the reconstruction. As explored in more detail in Appendix *A* [App appa], the standard ghost-imaging formula may be viewed as a superposition of approximately orthogonal functions. A direct consequence is that the rows of the measurement matrix **A** should obey an ‘approximate completeness relation’, which can be written as 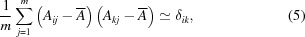
where 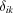 is the Kronecker delta. Note that, subtracting the average 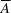 from each coefficient is required to have zero-mean terms, as each of the 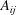 is non-negative on account of it being a measured intensity value. The previous equation would be exact only in the ideal case in which the measurement matrix (after subtracting its average) forms a complete orthonormal set.

To make the previous argument more apparent, let us explicitly rewrite the rows of the measurement matrix as the measured speckle images 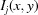, where 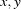 are the coordinates on the detector plane and the index *j* runs over the number of measurements. With this new, more transparent, notation the completeness relation in equation (5) can be rewritten as 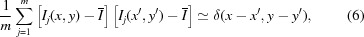
where 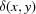 is the Dirac delta. As before, equation (6) is exact only when the speckle images subtracted by their average form a complete orthonormal set. In all practical cases, the previous equation can be used to instead define an effective point-spread function (PSF), 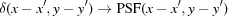 centred around the point 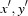 [see equation (14) in Appendix *A* [App appa], together with Ferri *et al.* (2010 ▸)].

**Figure 5:**
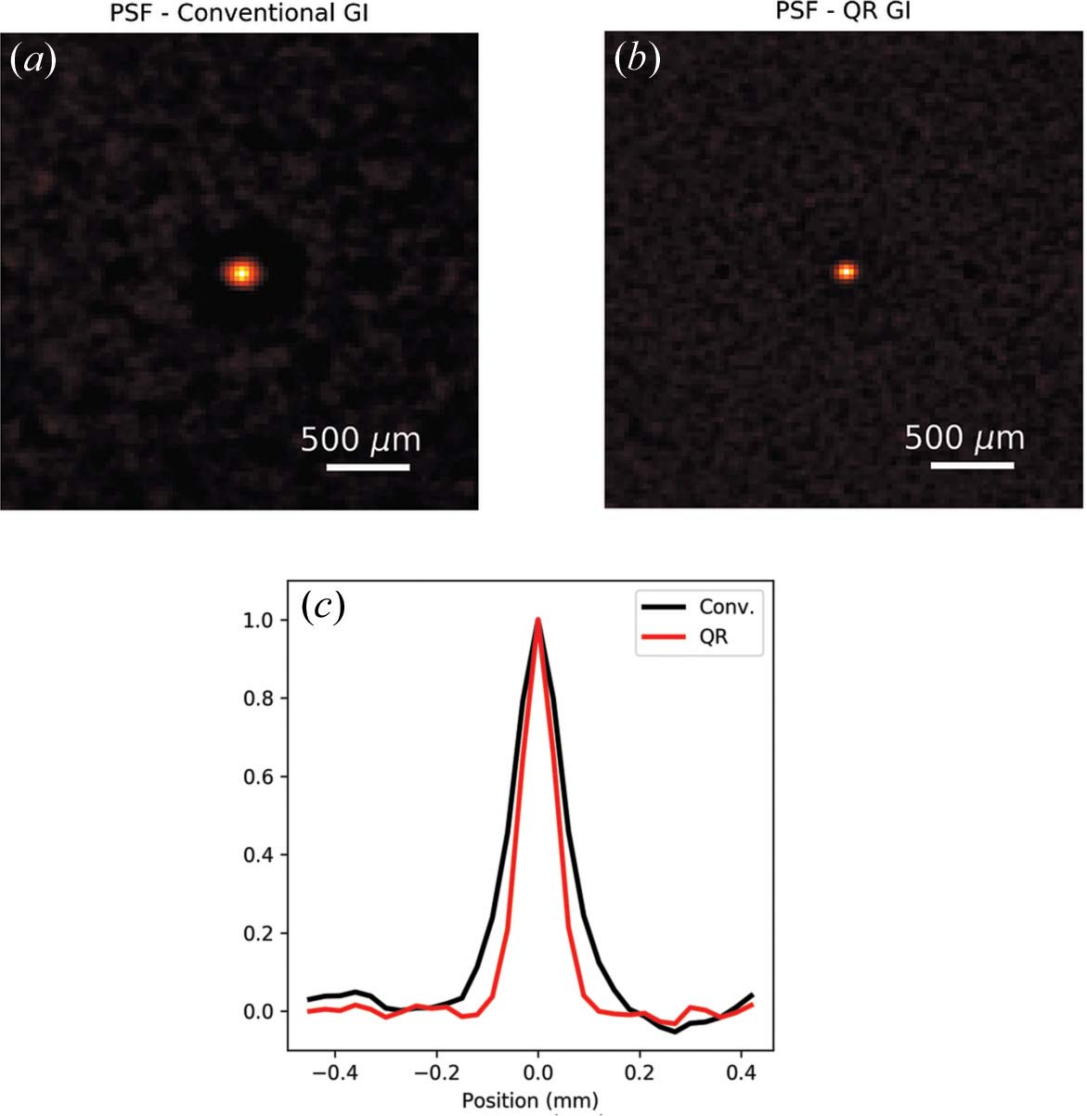
(*a*) PSF of the ghost-imaging system, calculated using equation (6), for the conventional ghost-imaging situation. (*b*) Corresponding PSF calculated after QR decomposition. The PSF appears noticeably narrower, reflecting the resolution improvement afforded by the QR decomposition. (*c*) Line profile taken across the central horizontal line in the maps in (*a*) black solid line and (*b*) red solid line. When fitted with a Gaussian function, the two peaks have a FWHM of 125 µm and 80 µm respectively.

This PSF governs the spatial resolution of the ghost-imaging system; the auto-covariance of the speckle fields defines the ghost-imaging PSF, which implies a spatial resolution comparable to the speckle size (for example, Oh *et al.*, 2013 ▸; Sprigg *et al.*, 2016 ▸). In light of this idea, we calculated equation (6) for the conventional ghost-imaging situation [where the 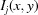 are the original speckle images] as well as for the modified speckle images after the QR decomposition. The results are shown in Fig. 5 ▸. When using the original mask, the full width at half maximum (FWHM) of the approximately rotationally symmetric PSF (fitted with a Gaussian) is about 125 µm, which reduces to about 80 µm after the QR decomposition.

The form of ghost imaging presented here, equipped with the definition of the PSF as discussed above, may be viewed as a form of scanning probe imaging (see, for example, Pennycook & Nellist, 2011 ▸) using a completely delocalized probe and a large integrating bright-field detector. While the resolution of scanning probe imaging is usually dictated by the size of a localized scanning probe, for our delocalized scanning probe the resolution is limited by the smallest characteristic length scale present in the intensity fluctuations of the ensemble of illuminating speckle fields [*cf.* equation (14) in Appendix *A* [App appa]], which is in turn the width of the PSF calculated using equation (6).

### 4.2. Other practical aspects of X-ray ghost imaging   

We have previously mentioned the robustness of ghost imaging (Meyers *et al.*, 2007 ▸, 2008 ▸, 2011 ▸, 2012 ▸; Hardy & Shapiro, 2011 ▸; Tajahuerce *et al.*, 2014 ▸). This robustness arises from the invariance of the ensemble average in equation (2), with respect to the addition of statistically uncorrelated fluctuations in the object and reference arms of the ghost-imaging setup. Stated more precisely, the ensemble average in equation (2) is unchanged under either or both of the replacements 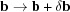 and 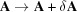, where 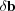 is a zero-average random fluctuation added to the bucket signal and 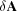 is a random fluctuation added to the speckle images, provided that 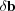 and 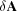 are not correlated. In our experimental case, this fact amounts to the statement that the ghost-imaging process is substantially robust against uncorrelated noise in the bucket or reference beam. We expect, however, that the vibration-induced blurring in the bucket beam discussed in §2, does have a negative effect on the speckle-to-speckle correlation and must, therefore, be minimized. Specifically, while the total number of photons incident on the sample is unaffected by the blurring (at least to a first approximation) the local distribution will cause the bucket signal to slightly change to the effect of reducing the excursion of the signal relative to its mean. This in turn, will make the image-formation process less efficient, *i.e.* the ghost image is less clear for the same number of frames *m*.

**Figure 6:**
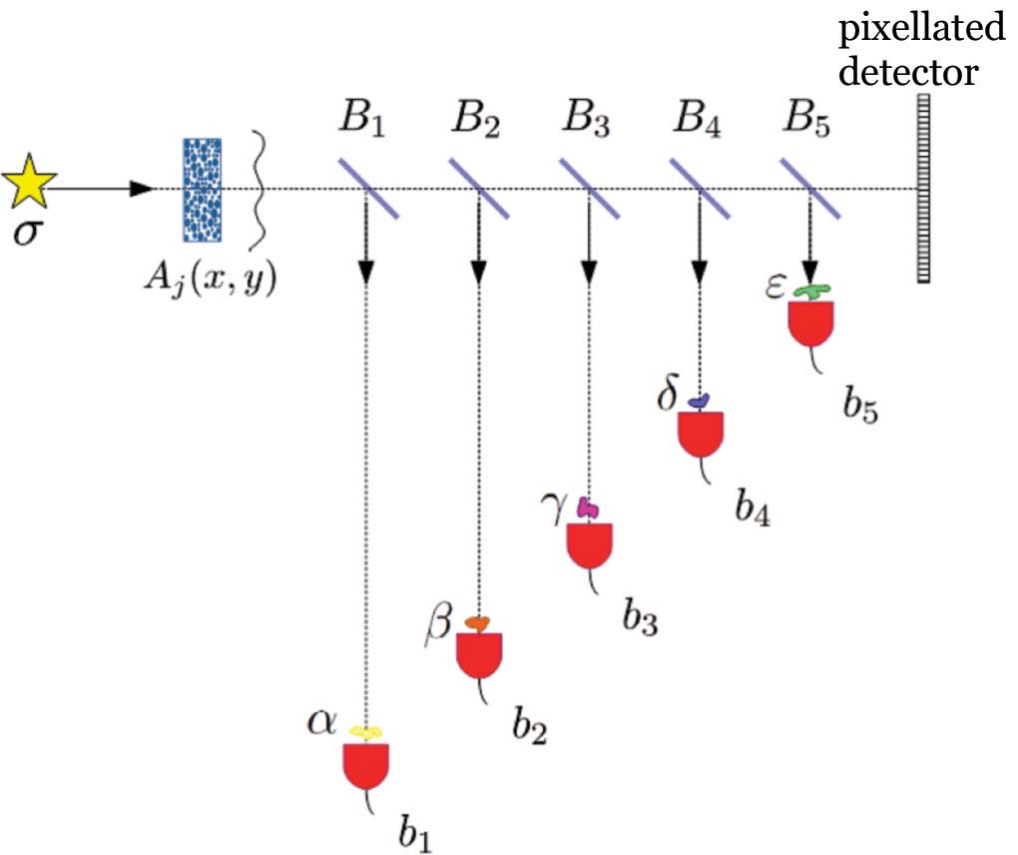
Schematic setup for parallelized X-ray ghost imaging.

Another practical aspect of X-ray ghost imaging in the experimental setup used here, is its ability to be parallelized. Inspired by the parallel form of computational ghost imaging proposed by Yuan *et al.* (2016 ▸), consider the setup shown in Fig. 6 ▸. Here, an X-ray source σ illuminates an ensemble of *m* random speckle-producing masks 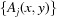, where 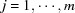 labels each realization of the mask and 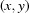 are coordinates in the plane perpendicular to the optic axis. A series of beamsplitters 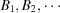 then illuminate a series of objects 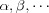, giving associated non-spatially resolved signals in the bucket detectors 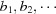 Each bucket signal in each detector may be correlated with the same ensemble of speckle images registered by the pixellated array detector for each realization of the mask, to yield independent parallelized ghost imaging. The objects in Fig. 6 ▸ are staggered so as to keep constant the source-to-object distance, thereby ensuring that Fresnel diffraction and other free-space-propagation effects are accounted for, with the registered speckle pattern measured over the pixellated array detector being equal (up to a multiplicative constant) to the speckle patterns illuminating each object. Note that each beamsplitter only needs to remove a negligible fraction of the total energy from the beam which ultimately illuminates the pixellated array detector; the resulting attenuation of the speckle-basis images registered by the array detector can be trivially taken into account in the parallel ghost reconstructions. Note also that, while Fig. 6 ▸ indicates one object per beamsplitter, one could also have multiple objects per beamsplitter, using multiple Bragg or Laue reflections from a crystal beamsplitter, or multiple Laue reflections from a polycrystal beamsplitter.

Despite the interesting avenues offered by parallelized ghost imaging, we should note that the use of a crystal beamsplitter is not ideal, with the current stage of technology at least. Indeed, since the object-plus-bucket beam need only be very weak, a crystal beamsplitter is in no way essential for the realization of X-ray ghost imaging. For example, while the experiment of Schori & Schwartz (2017 ▸) uses a pyrolytic graphite crystal as a beamsplitter, the experiment of Zhang *et al.* (2018 ▸) needs no beamsplitter at all since the object-free reference images are pre-recorded. This latter case is a close analogue of the concept of computational ghost imaging in the optical domain.

On this note, in the spirit of computational (ghost) imaging (Shapiro, 2008 ▸; Bromberg *et al.*, 2009 ▸; Sun *et al.*, 2013 ▸), one may even dispense altogether with the pixellated array detector. To do this, one would have a highly structured mask whose three-dimensional micro-structure is so well characterized, and the illuminating beam so stable and well characterized, that one could use a numerical implementation of the X-ray scattering and diffraction to calculate the ensemble of reference speckle fields that one would have measured had an array detector been used; therefore, these images do not need to be measured. One would then have a form of computational X-ray ghost imaging using only bucket detectors. In this context, we point out that the function played by spatial light modulators in visible-light computational imaging is replaced with the known micro-structured mask, in the proposed form of X-ray computational imaging. In the near future, this highly structured mask might even be amenable to fabrication using 3D printing technology.

In addition, we note that spatially random masks are not necessarily optimal for X-ray ghost imaging. While random masks are often easy to synthesize using, for example, spatially disordered condensed matter, other structures such as the uniformly redundant array (Fenimore & Cannon, 1978 ▸) may be more efficient.

Regarding the ultimate resolution that is achievable in principle by ghost imaging, the preceding analysis makes clear that this equates to the smallest speckle size that may be achieved using X-rays. Since the smallest X-ray speckle size that may be attained is itself limited by the wavelength of the X-rays, it is the X-ray wavelength that governs the ultimate resolution achievable by ghost imaging. Thus, for example, one could return to the use of shot noise to generate speckles (Pelliccia *et al.*, 2016 ▸), work in a geometry where the reference beam is magnified, and recall that the object-plus-bucket beam does not influence resolution. In this X-ray ghost microscopy scenario, which is a minor extension of that previously demonstrated by Pelliccia *et al.*, (2016 ▸), the resolution would be limited by the resolution with which a magnified shot-noise image could be detected.

## 5. Conclusions   

We have presented an experimental realization of X-ray ghost imaging using synchrotron X-rays from an undulator. This demonstration has been developed to explore practical avenues for producing X-ray ghost images. We reported the measurements of two samples, a stencil in a lead mask and a tungsten coil. For both samples we reported three different reconstruction strategies. The first is based on the conventional ghost-imaging formula, in which the ghost image is approximated by the weighted average of the speckle illuminating images. The weights of the superposition are the bucket signals subtracted by their average. The second approach is based on prior QR decomposition of the measured speckle reference images. In this way, the ensemble of speckle images can be made to be a better approximation to an orthogonal basis, thereby improving the resolution of the reconstruction. The third approach uses Landweber iteration to refine the ghost reconstruction still further. Next, we analysed in more detail the resolution of our ghost-imaging system, defining an effective PSF which was shown to be improved upon QR decomposition of the illuminating functions. Finally, we discussed practical aspects for future applications of X-ray ghost imaging, including its robustness against uncorrelated intensity fluctuations, and improved measurement strategies using parallelized ghost imaging and computational X-ray ghost imaging.

## Appendix A Estimating ghost-imaging resolution from a given speckle basis

Consider an ensemble of  spatially random two-dimensional intensity speckle patterns  defined over a domain Ω with area  in the  plane. Each ensemble member is labelled by the integer *j*, with each  being non-negative on account of being an intensity map.

Assume ‘many speckles’ in each random speckle field. More precisely, consider each to be a distinct realization of a stochastic process with every member of the ensemble of speckle fields having the same characteristic transverse length scale σ, in both *x* and *y*. Stated differently, we assume each realization of the speckle field to be statistically identical. Since each speckle field has *M* speckles with , this implies that (i) the spatially averaged intensity of each realization of the ensemble is approximately the same; (ii) the spatially averaged squared intensity, of each member, is also approximately the same; and (iii) the ensemble-averaged intensity at any point in Ω is independent of position, and approximately equal to the spatially averaged intensity in any particular realization.

The standard ghost-imaging formula considers the ensemble  as a speckle basis, from which the ghost image of the object transmission function  may be synthesized (Bromberg *et al.*, 2009 ▸; Katz *et al.*, 2009 ▸),

where, * denotes convolution over *x* and *y*,  is a point-spread function associated with the finite spatial resolution with which  is estimated, the bucket signal is

and the average bucket signal is

Using the above definitions and assumptions, one can readily show that

where

and

Using equations (8), (10) and (11), equation (7) can be manipulated into the form:

Since *v*(*x*,*y*)*PSF(*x*,*y*) = , the point-spread function associated with the ghost-imaging reconstruction is the ensemble-averaged intensity–intensity correlation between the locations  and  in the illuminating speckle fields (Ferri *et al.*, 2010 ▸), corresponding to the smoothed completeness relation:

For any fixed position , and a specified measured ensemble of intensity-speckle fields, equation (14) can be used to estimate the local resolution associated with superpositions [such as equation (7)] that utilize this basis in a ghost-imaging context – see Fig. 5 ▸. The above expression may also be used to generate a ‘pixel basis’ consisting of a lattice of PSFs whose centroids are separated by the FWHM of each PSF. This set of PSFs is another approximately orthogonal basis, which is complementary to the approximately orthogonal speckle basis from which it is derived, insofar as the former is localized whereas the latter is not. Indeed, one may loosely speak of equation (14) as showing how linear combinations of the spatially delocalized speckle probes may be formed to yield very localized probes.

Interestingly, a variant of the above chain of reasoning may be used to derive the standard ghost-imaging formula from first principles, in a physically transparent manner. One can start with an ensemble of intensity speckle fields that obey the previously articulated assumptions (see the second paragraph of this Appendix). The intensity covariance on the right hand side of equation (14) will typically be a narrowly peaked normalizable function, making it natural to adopt it as a (possibly position-dependent) point-spread function, by definition. The convolution of this point-spread-function with a well behaved but otherwise arbitrary function , then leads directly to the standard ghost-imaging formula in equation (7), by reversing the logical development of this Appendix.

We close by noting that the standard ghost-imaging formula may be viewed in approximate terms as a form of orthogonal function expansion. Indeed, for our ensemble of speckle images, each of which are statistically identical and each of which contain many speckles, symmetry considerations dictate the following expression for the (approximate) orthogonality of distinct background-subtracted speckle fields:

where

and  is the variance of each , which is assumed to be independent of *j* on account of the previously articulated assumptions. By construction, the set  of speckle patterns has each member averaging to zero over Ω, with distinct members being orthogonal to each another, and all members being approximately orthogonal to a constant offset. Thus, the standard ghost-imaging formula, as a weighted superposition of the near-orthogonal set of functions , may be viewed in approximate terms as a form of orthogonal function expansion. Such near-orthogonality of distinct background-subtracted speckle fields evidently forms a natural albeit implicit assumption when using equation (1) directly.

## Appendix B Details on ghost-imaging reconstructions using QR decomposition

The QR decomposition approach stems from the observation that equation (1) is, strictly speaking, exact only when the speckle basis is exactly orthonormal. This is the case reproduced in visible-light single-pixel cameras using a measurement matrix equal to the Hadamard matrix . In this case, redefining the bucket signal as , in the absence of noise, the expansion in equation (1) is exact when . While this is a convenient choice in the visible part of the spectrum (where efficient spatial light modulators exist), this option is not currently easy to implement for X-ray imaging.

The QR decomposition is a solution to this issue. Starting from the measurement matrix  describing the speckle basis, we can generate an orthogonal matrix by QR decomposition of :

where,  is the orthogonal matrix we seek, and  is an upper triangular matrix [*cf.* the related more sophisticated approach, utilizing Moore–Penrose pseudo-inverses, by Zhang *et al.* (2014 ▸)].

Once the decomposition is obtained, the only non-trivial step is to rearrange the bucket vector  accordingly. Specifically, we seek a vector , that is the bucket signal that would be measured if the measurement matrix were the orthogonal matrix . Since , we can write

Therefore, by inverting the previous expression:

Equations (17) and (19) constitute the algorithm we use on our data to produce an approximately orthonormal decomposition. The new measurement matrix  and the transformed bucket signal  can be used in equation (1) to obtain the ghost-imaging reconstruction.
